# p53-dependent G2 arrest associated with a decrease in cyclins A2 and B1 levels in a human carcinoma cell line

**DOI:** 10.1054/bjoc.1999.0976

**Published:** 2000-01-18

**Authors:** C Badie, J Bourhis, J Sobczak-Thépot, H Haddada, M Chiron, M Janicot, F Janot, T Tursz, G Vassal

**Affiliations:** Laboratoire de Pharmacotoxicologie et Pharmacogénétique UMR8532, Laboratoire UPRES EA N°27-10 ‘Radiosensibilité-Radiocarcinogénèse humaine' Institut Gustave-Roussy, 94805 Villejuif Cedex, France; Unité Inserm 370, Faculté Necker, 156 rue Vaugirard, 75730 Paris Cedex 15, France; U362, Inserm; Rhone-Poulenc Rorer Gencell, Vitry/Seine, France

**Keywords:** p53 gene transfer, cell cycle arrest, cyclius, adenovirus vector

## Abstract

In vivo transfer of wild-type (wt) p53 gene via a recombinant adenovirus has been proposed to induce apoptosis and increase radiosensitivity in several human carcinoma models. In the context of combining p53 gene transfer and irradiation, we investigated the consequences of adenoviral-mediated wtp53 gene transfer on the cell cycle and radiosensitivity of a human head and neck squamous cell carcinoma line (SCC97) with a p53 mutated phenotype. We showed that ectopic expression of wtp53 in SCC97 cells resulted in a prolonged G1 arrest, associated with an increased expression of the cyclin-dependent kinase inhibitor WAF1/p21 target gene. A transient arrest in G2 but not in G1 was observed after irradiation. This G2 arrest was permanent when exponentially growing cells were transduced by Ad5CMV- *p53* (RPR/INGN201) immediately after irradiation with 5 or 10 Gy. Moreover, levels of cyclins A2 and B1, which are known to regulate the G2/M transition, dramatically decreased as cells arrived in G2, whereas maximal levels of expression were observed in the absence of wtp53. In conclusion, adenoviral mediated transfer of wtp53 in irradiated SCC97 cells, which are mutated for p53, appeared to increase WAF1/p21 expression and decrease levels of the mitotic cyclins A2 and B1. These observations suggest that the G2 arrest resulted from a p53-dependent premature inactivation of the mitosis promoting factor. © 2000 Cancer Research Campaign


British Journal of Cancer (2000) **82**(3), 642–650
© 2000 Cancer Research Campaign
DOI: 10.1054/ bjoc.1999.0976, available online at http://www.idealibrary.com on IDEAL®

### p53-dependent G2 arrest associated with a decrease in cyclins A2 and B1 levels in a human carcinoma cell line

**C Badie[1,*], J Bourhis[2], J Sobczak-Thépot[3], H Haddada[4], M Chiron[5], M Janicot[5], F Janot[1], T Tursz[1] and G Vassal[1]**

[1]Laboratoire de Pharmacotoxicologie et Pharmacogénétique UMR8532 [2]Laboratoire UPRES EA N°27-10 'Radiosensibilité-Radiocarcinogénèse humaine' Institut Gustave-Roussy, 94805 Villejuif Cedex, France; [3]Unité Inserm 370, Faculté Necker, 156 rue Vaugirard, 75730 Paris Cedex 15, France, [4]U362, Inserm, [5]Rhone-Poulenc Rorer Gencell, Vitry/Seine, France

**Summary** In vivo transfer of wild-type (wt) p53 gene via a recombinant adenovirus has been proposed to induce apoptosis and increase radiosensitivity in several human carcinoma models. In the context of combining p53 gene transfer and irradiation, we investigated the consequences of adenoviral-mediated wtp53 gene transfer on the cell cycle and radiosensitivity of a human head and neck squamous cell carcinoma line (SCC97) with a p53 mutated phenotype. We showed that ectopic expression of wtp53 in SCC97 cells resulted in a prolonged G1 arrest, associated with an increased expression of the cyclin-dependent kinase inhibitor WAF1/p21 target gene. A transient arrest in G2 but not in G1 was observed after irradiation. This G2 arrest was permanent when exponentially growing cells were transduced by Ad5CMV-*p53* (RPR/INGN201) immediately after irradiation with 5 or 10 Gy. Moreover, levels of cyclins A2 and B1, which are known to regulate the G2/M transition, dramatically decreased as cells arrived in G2, whereas maximal levels of expression were observed in the absence of wtp53. In conclusion, adenoviral mediated transfer of wtp53 in irradiated SCC97 cells, which are mutated for p53, appeared to increase WAF1/p21 expression and decrease levels of the mitotic cyclins A2 and B1. These observations suggest that the G2 arrest resulted from a p53-dependent premature inactivation of the mitosis promoting factor. © 2000 Cancer Research Campaign

**Keywords:** p53 gene transfer, cell cycle arrest, cyclius, adenovirus vector

The p53 gene is probably the most frequently altered (mutation, deletion) tumour suppressor gene in human cancer cells (Friend, 1994; Sherr, 1996). It also plays a key role in the regulation of apoptosis and G1 arrest after treatment with DNA-damaging agents such as irradiation. The critical biochemical function of wild type (wt) p53 is its ability to bind to specific DNA sequences and to activate the transcription of genes such as GADD45 (Growth Arrest after DNA Damage), Bax involved in the regulation of apoptosis and p21 WAF1/CIP1 gene, whose product inhibits several cyclin/cyclin-dependent kinases complexes, essential for cell cycle progression (Deng et al, 1995). Ionizing radiations induce a delay both in G1 and G2. However, only the G1 delay appears to depend on p53 (Kastan et al, 1991). Hence most of the transformed and cancer cells which exhibit a mutated p53 fail to arrest in G1 after irradiation but still undergo a G2 arrest (Kastan et al, 1991).

The mechanisms underlying the radiation-induced G2 arrest are not fully understood. The progression of cells through G2 and M phases is regulated in eukaryotes by MPF (mitosis promoting factor) which includes two proteins, a cyclin (cyclin B) and a cyclin-dependent kinase (cdk) $p34^{cdc2}$ (cdk1). Another complex, cyclin A2–cdk1, is activated before cyclin B–cdk1 and is also required for the progression from G2 to mitosis. The kinase activity of cdk1 depends on both its association with the cyclin subunit and phosphorylation of specific residues. Levels of cdk1 remain relatively constant throughout the cell cycle, whereas levels of cyclins A and B fluctuate with a maximum in M phase (Sherr, 1996). Irradiation has been shown in mammalian cells to induce a decrease in cdk1 activity, related to a decrease of cyclin B1 and/or to an inhibition of cdk1 phosphorylation (Jin et al, 1996). Indeed cyclin B1 is a rate-limiting component of the radiation-induced G2 delay (Kao et al, 1997). Down-regulation of cyclin B1 occurs at least in part by a post-transcriptional regulation of mRNA levels (Maity et al, 1995). In addition, Azzam et al (1997) showed in various cell lines that cdk1 is down-regulated by γ-radiation through a p53-dependent mechanism.

Several studies have recently suggested that wtp53 might also regulate the cell cycle arrest in G2/M after DNA damage (Agarwal et al, 1995; Allday et al, 1995; Aloni-Grinstein et al, 1995; Guillouf et al, 1995; Han et al, 1995; Powell et al, 1995; Stewart et al, 1995; Pellegata et al, 1996; Skladanowski et al, 1997). However, the contribution of p53 seems to be complex and to depend on the cell type. Most studies were performed in rodent cells and relatively little is known about this effect in human tumour cell lines, although a recent study by Hermeking et al (1997) has shown a p53-regulated inhibition of G2M progression in a human rectal carcinoma model. In addition, none of these studies has investigated the effect of p53 expression on the levels of cyclins A2 and B1 during the G2 arrest.

In our study we further examined the consequences of irradiation combined to the expression of wtp53 after gene transfer on (i) the radio-induced G2 arrest, (ii) expression of the mitotic cyclins

*Received 24 February 1999*
*Revised 7 September 1999*
*Accepted 14 September 1999*

*Correspondence to:* J Bourhis, Laboratoire UPRES Radiosensibilité-Radiocarcinogénèse humaine, Institut Gustave Roussy, 39 rue Camille Desmoulins, 94805 Villejuif cedex, France.

*Present address: Gene Targeting Group, MRC Clinical Sciences Centre, Imperial School of Medicine, Hammersmith Hospital, Du Cane Road, London W12 0NN, UK

A2 and B1, and (iii) apoptosis. Since this study was designed in the context of combining p53 gene therapy and irradiation, we have used a recombinant adenovirus to transfer the wtp53 gene in a head and neck squamous cell carcinoma line SCC97, which exhibited a mutated p53 phenotype. An adenoviral vector was used since it ensures a particularly high gene transfer efficiency, and is commonly used in most gene therapy studies (Gallardo et al, 1996; Clayman et al, 1996).

### MATERIALS AND METHODS

### Cell line

The study has been performed using the human squamous cell carcinoma cell line SCC97 (gift of G Clayman, MD Anderson, Houston, TX, USA). Cells were grown in modified essential medium (MEM) supplemented with 15% fetal calf serum (FCS) with penicillin–streptomycin and 2 mM glutamine at 5% carbon dioxide. The clonogenic survival curve after γ-irradiation has been established and the surviving fraction at 2 Gy was 59%.

SCC97 cells exhibit a mutation of p53 gene, that was characterized by sequencing. A point mutation (transversion T–A) at the second base of the fifth intron inactivates the normal splicing donor site. An alternative splicing therefore occurs between a GT sequence in exon 5 as a donor site and the usual acceptor site. This results in a 46 bp deletion in the coding sequence in exon 5 and a frame-shift, leading to a truncated unstable p53 which cannot be detected by immunofluorescence or Western blot. Western blot analysis and flow cytometry have been performed using DO7 and DO1 monoclonal antibodies against the N-terminal part of the human p53 protein, showing that the mutated p53 protein was not detectable.

### Recombinant adenovirus

A human non-replicative recombinant adenovirus type 5, with a complete deletion of E1A and a partial deletion of E3 regions was used as vector to infect the cell line in vitro. This vector encoded the human wtp53 gene driven by the human cytomegalovirus (CMV) promotor. A batch was provided by RPR-Gencell (Vitry, France, Ad5CMV-*p53* RPR/INGN201). The construction and properties of Ad5CMV-*p53* have been reported elsewhere (Zhang et al, 1994). The vector AV1.0CMV/293 was used as a control. Viruses were propagated, purified, titrated in 293 cells transcomplementing for E1 gene products, and assessed for the absence of contaminating wild-type recombinant virus using protocols previously described (Zhang et al, 1995). Viral titre was determined by UV-spectrophotometric analysis (viral particle $ml^{-1}$) and by plaque forming assay (pfu $ml^{-1}$). Titres of the viral stocks used were between $10^9$ and $10^{11}$ pfu $ml^{-1}$. Viruses were stored at –80°C.

### Gene delivery and fluorescence-activated cell sorter analysis

Viruses were diluted in Dulbecco’s MEM (DMEM) supplemented with 2% FCS in polypropylene tubes. In vitro transduction were performed by plating $1\times10^6$ cells in 75-$cm^2$ flasks. Twenty-four hours after plating, cells were incubated for 1 h at 37°C with purified virus in 6 ml of MEM supplemented with 10% FCS. At various times after infection, cells were trypsinized and fixed by the addition of a formaldehyde solution (4% in phosphate-buffered saline (PBS)) for 20 min at room temperature. Cells were pelleted and permeabilized in 0.2% Triton X-100 in PBS for 10 min at room temperature. After three rinses in Power Block 1X (Biogenex), cells were incubated with the anti-p53 antibody (p53-FITC DO7, 1/200) (Dako) for 2 h at room temperature. Cells were rinsed twice in Power Block and then stored at 4°C until analysed. A control was done by incubating cells with a mouse IgG1 fluorescein isothiocyanate (FITC) (1/100) (Becton Dickinson) to estimate the amount of non-specific binding of mouse monoclonal antibodies.

### p53 mRNA expression

Total RNAs were extracted from SCC97 cells and mRNAs were purified with the QuickPrep Micro mRNA Purification Kit (Pharmacia Biotech). To determine the p53 phenotype of the cells, a functional test was performed in yeast, which reveals the loss of the transactivating function of the mutated p53 protein (Flaman et al, 1995). Briefly, p53 mRNAs were reverse transcribed and amplified by polymerase chain reaction (PCR). The *Saccharomyces cerevisiae* strain yIG397 was co-transformed with the reverse transcription PCR (RT-PCR) product and a linearized expression vector (pSS16) carrying the 5′ and 3′ ends of the p53 open reading frame and the LEU2 gene. Yeast colonies in which a homologous recombination between the linearized pSS16 and the PCR product had occurred expressed constitutively p53 and were selected on medium lacking leucine. The strain used also contains an integrated plasmid with the ADE2 open reading frame under the control of a p53-responsive promoter. Yeasts transformed with a plasmid encoding wtp53 grew normally and formed white colonies (ADE2+). Yeasts expressing mutant p53 failed to express ADE2 and formed small red colonies because adenine is limiting for cell growth (phenotype $ADE2^-$).

### Western blot analysis

For Western blot analysis, total cellular proteins were isolated by lysing the cells in 50 mM Tris–HCl pH 7.5, 50 mM sodium chloride, 1% NP-40, 0.5% sodium deoxycholate, 0.1% sodium dodecyl sulphate (SDS) and a protease inhibitor cocktail (Boehringer Mannheim). Protein concentrations were determined by the micro bacteriochlorine *a* (BCA) protein protein assay reagent kit (Pierce). Proteins (50 μg) were separated on 9% (to detect p53, cyclins A2 and B1) or 12% (to detect p21/WAF1) SDS-polyacrylamide gels and electroblotted to Hybond-C membranes (Amersham*). After the transfer was completed, the membranes were stained with Ponceau S (Sigma*) to verify equal sample loading. Membranes were probed with antibodies directed against cyclin A2 (monoclonal 11B2G3, Dr Joëlle Sobczak-Thépot), cyclin B1 (H433, SantaCruz Biotechnology), p53 (DO7, Dako) and p21/WAF1 (Ab1, Oncogene Research Biotechnology Products). These primary antibodies were diluted at 1/20 000, 1/10 000, 1/1000 and 1/500 respectively. The bound antibodies were detected by enhanced chemiluminescence (ECL) using an ECL kit (Amersham*). Relative levels of cyclin A2 and B1 were determined by scanning the blots.

© 2000 Cancer Research Campaign

### Analysis of apoptosis and cell cycle

Detection of apoptotic cells by flow cytometry was performed using the in situ cell death detection kit, fluorescein by TUNEL (terminal deoxynucleotidyl transferase (TdT)-mediated dUTP nick end labelling, Boehringer Mannheim). The TUNEL assay was performed using a procedure previously described. Briefly, cells were trypsinized and centrifuged with the culture medium to pellet in the same tube attached cells and cells in suspension. Cells were washed in bovine serum albumin (BSA) solution (1% in PBS) and fixed in 4% paraformaldehyde (in PBS, pH 7.4) for 30 min at room temperature. Cells were pelleted and permeabilized with 0.3% Triton X-100 in sodium citrate 0.1% for 2 min on ice. Cells were then rinsed twice in PBS and incubated with the TUNEL reaction mixture. A negative control was done by incubating cells in the reaction mixture without terminal transferase and a positive control was provided by incubating cells with DNAase 1 (Boehringer Mannheim, 50 mg $ml^{-1}$ in PBS, for 10 min at room temperature) prior to the TUNEL reaction. Cells were then washed twice in PBS and incubated for 30 min at 37°C with 10 mg $ml^{-1}$ RNAase and with propidium iodide at 500 mg $ml^{-1}$. Two-colour fluorescence analysis was done on a fluorescence-activated cell sorter (FACS).

Cells analysed for their DNA content were fixed in ice-cold 70% ethanol and stored at –20°C until analysis. For FACS analysis, cells were resuspended in PBS and stained with propidium iodide as described above.

### Cell synchronization

Cells were incubated with 200 μM mimosine (Sigma) which is a plant amino-acid for 24 h to synchronize them in G1/S.

### Irradiation

Cells were irradiated with a 137Cs γ-ray source at a dose rate of 1.45 Gy $min^{-1}$ at room temperature, in culture dishes.

### RESULTS

### Expression of wtp53 after adenovirus-mediated gene transfer

Expression of wtp53 mRNA in SCC97 cells infected with Ad5CMV-*p53* was estimated using the yeast functional test (Flaman et al, 1995). The percentage of white yeast colonies reflected the ratio between the ectopic mRNA encoding wtp53 and the endogeneous mRNA encoding mutant p53. We found 90% of white colonies starting with mRNAs purified from SCC97 cells 48 h after infection with Ad5CMV-*p53*. This value was independent on the multiplicity of infection (MOI) in the range of 25–800 MOI. Using the same assay, we found that expression of wtp53 was already detectable 2 h after infection and reached a plateau 4 h after infection (Figure 1A). These results indicated that wtp53 mRNA was efficiently expressed from the viral vector.

The proportion of cells expressing the protein wtp53 was then determined by FACS analysis as a function of the MOI (Figure 1B, left). A maximum of 90% of wtp53-positive cells was observed at 100 MOI. Therefore, we subsequently used this MOI to assess the effect of wtp53 in the whole cell population, expressing wtp53 relatively homogeneously. The wtp53 protein was rapidly expressed after infection since it was detectable by flow cytometry

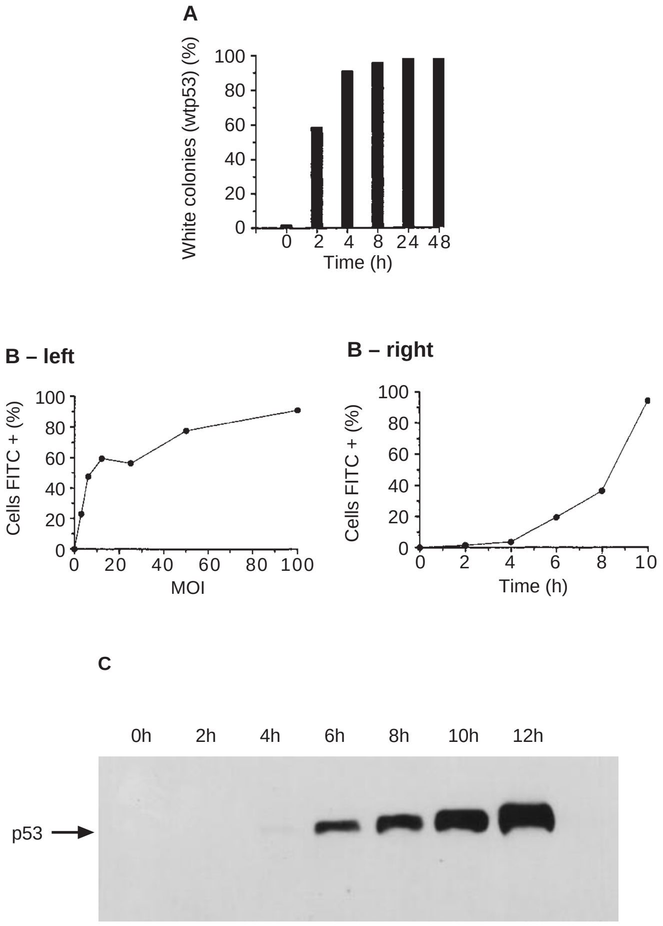

**Figure 1** (**A**) Expression of exogenous wild-type p53 mRNA in SCC97 cells after infection with Ad5CMV-*p53* at 25 MOI. The results are expressed as the proportion (%) of white/total yeast colonies. (**B**) Expression of wtp53 mediated by Ad5CMV-*p53* in SCC97: transduction efficiency analysis obtained by immunofluorescence cytometry in SCC97 cells at different MOI (**B** – left) and different times after infection with 100 MOI of Ad5CMV-*p53* (**B** – right). The percentages of p53-positive cells were obtained by flow cytometry. (**C**) Time course of p53 protein expression after Ad5CMV-*p53* (100 MOI) infection. The blots are shown with the time in hours after infection indicated above the lanes. After cell lysis, the proteins, separated by SDS-PAGE, were transferred to a membrane and probed with antibodies recognizing p53 (DO7). The band observed corresponded to wtp53 since the endogenous p53 truncated protein in SCC97 was not recognized by the antibody we employed

4 h after infection and was maximal in 90% of cells after 10 h (Figure 1B, right). In addition, Western blot analysis revealed that a band corresponding to wtp53 was detected at 4 h and that the amount of wtp53 still increased after 10 h (Figure 1C).

### Combined effects of wtp53 gene transfer and irradiation on the cell cycle

Cell cycle alterations have been studied up to 48 h after infection with Ad5CMV-*p53* and/or irradiation (Figure 2A). Adenovirus-mediated gene transfer of wtp53 induced a cell cycle arrest in the G1 phase in 70% of cells 24 h after infection (data not shown). This arrest persisted at 48 h (Figure 2A) and 72 h after infection with a percentage of cells in S phase below 10%. This arrest was p53-dependent since cells infected with the control vector

© 2000 Cancer Research Campaign

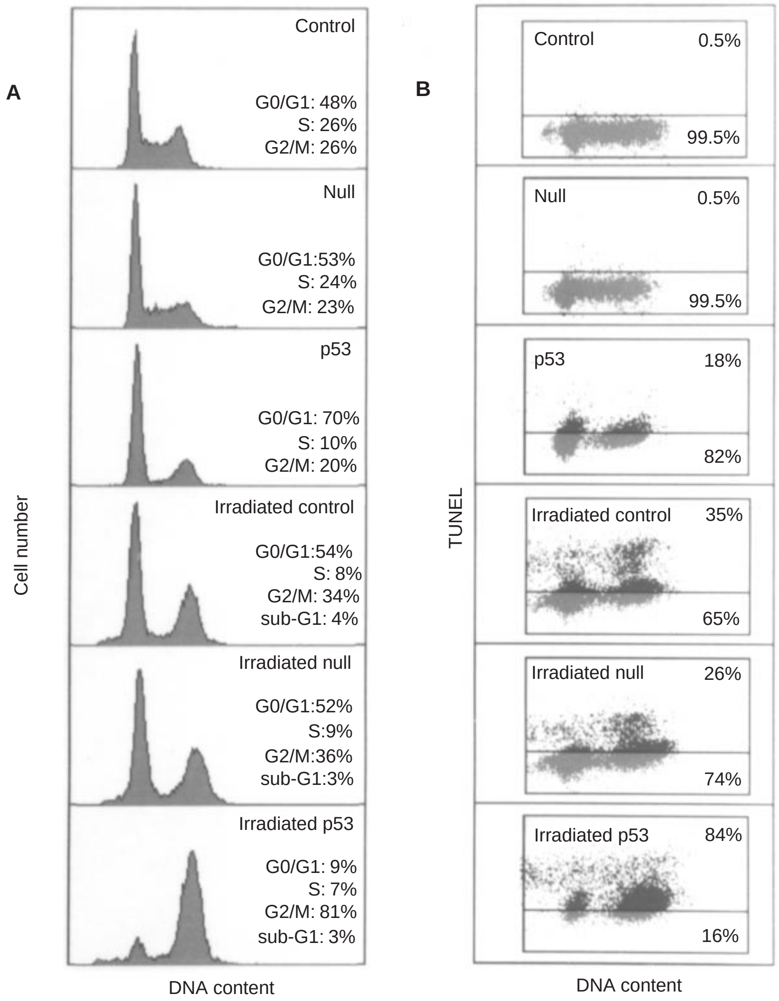

**Figure 2** (**A**) Cell cycle analysis, 48 hours after treatment for control, cells infected by the control vector AV1.0CMV/293 alone (null) or associated with irradiation (10 Gy), cells infected by Ad5CMV-*p53* alone or associated with irradiation and irradiation alone. Propidium iodide stained cells were analysed by FACS. The percentage of cells in each phase of the cell cycle (subG1, G1, S and G2/M) is shown in each panel. (**B**) Labelling of DNA breaks in apoptotic cells with FITC dUTP by TUNEL method. After infection, flow cytometric analysis for apoptosis was performed at 48 h. The percentage of cells in apoptosis is shown in the upper part of each panel. Two-colour fluorescence analysis was done on FACS: a linear gate was drawn to include all cells within a cell cycle propidium iodide fluorescence profile and then projected onto a scatter plot of logarithmic FITC signal versus propidium iodide integral signal of fluorescence

AV1.0CMV/293 were not arrested and maintained an exponential growth.

Irradiation (5 or 10 Gy) of SCC97 cells induced predominantly a cell arrest in G2 but not in G1. This G2 arrest was reversible and had almost completely disappeared 48 h after irradiation (Figure 2A). In contrast, when cells were infected with Ad5CMV-*p53* immediately after irradiation at 2.5, 5 (data not shown) or 10 Gy, the G2 arrest was still observed 48 h after irradiation (Figure 2A). In addition, the fraction of cells blocked in G2 was dose-dependent: 21% for 2.5 Gy, 41% for 5 Gy and 81% for 10 Gy. This protracted G2 arrest was p53-dependent since cells transduced with the control vector AV1.0CMV/293 showed a transient G2 arrest as uninfected irradiated cells (Figure 2A).

### Apoptosis

The occurrence of apoptosis after p53 gene transfer and/or irradiation was studied by the TUNEL assay followed by flow cytometry 24 h (data not shown) and 48 h after treatment (Figure 2B). The cell cycle was analysed in parallel. Almost no apoptosis was detected in control uninfected cells (0.5%) and in cells infected with the control vector AV1.0CMV/293. When these cells were irradiated (10 Gy), or infected by the control adenovirus, apoptosis occurred in both cases in 26–35% of cells (Figure 2B). Infection with Ad5CMV-*p53* induced apoptosis in 18% of cells, equally distributed in G1 and G2 phases of the cell cycle. This value increased to 84% when infection with Ad5CMV-*p53* was performed after irradiation. These apoptotic cells were in the G2 phase for most of them. This was a large scale effect since no cells remained attached to the culture dishes 72 h after irradiation and infection. In conclusion, these results showed that the G2 arrest induced by irradiation which was prolonged when combined to wtp53 gene transfer, was rapidly followed by a massive apoptosis. In contrast, when cells were infected first by Ad5CMV-*p53* and subsequently irradiated, a cell cycle arrest in the G1 phase was observed, with no significant increase of apoptosis (data not shown).

*© 2000 Cancer Research Campaign* 

### Expression of p21, cyclin A2 and cyclin B1 in synchronized cells after wtp53 gene transfer

To study the mechanisms underlying the cell cycle arrest after irradiation associated with Ad5CMV-*p53* infection, we used a homogeneous cell population synchronized in G1/S. Figure 3 shows that SCC97 cells were efficiently synchronized in G1/S upon treatment with 200 mM mimosine, with 89% of cells arrested at the G1/S boundary (T0 which represents the time of mimosine removal). Fourteen hours after release from the mimosine block, almost all the cells were in late S phase and in G2. Finally, 20 h after drug release, most of the cells were in G2 and a small percentage (11%) of them were already in G1 phase of the next cell cycle.

Irradiation of cells at the time of mimosine release (T0) did not inhibit S phase entry but induced a delay in G2, that was clearly observed at $t = 16$ h. At $t = 20$ h, only a few proportion of cells had escaped the delay in G2 and were in G1, compared to the control non irradiated cells (data not shown).

Western blot analysis showed that in control non irradiated cells the expression of cyclins A2 and B1 peaked 14 h and 16 h after release from the mimosine block respectively, corresponding to cells in late S and G2 phases (data not shown). After irradiation (5 Gy), the maximal expression of both cyclins was delayed and occurred between 12 and 22 h (cyclin A2) and peaked at 20 h (cyclin B1) (Figure 4). Maximal expression of these mitotic cyclins was therefore coincident with the delay in G2.

The expression patterns of cyclin A2 (Figure 4A) and cyclin B1 (Figure 4B) after irradiation (5 Gy) were then compared with or without wtp53 expression. The level of expression of cyclins A2 and B1 were dramatically reduced in cells infected with wtp53, as compared to non-infected cells (Figure 4A and B) or cells infected with the control vector (data not shown), and decreased to undetectable levels at 20–22 h. To determine if this effect was a direct consequence of wtp53 expression, the same experiment was repeated without irradiation. Figure 5 shows that the expression of wtp53 alone was sufficient to decrease the expression of both cyclins A2 and B1, without irradiation.

Finally, Figure 6 shows that p21/waf1 protein detected by Western blot was present only after expression of wtp53 protein and that irradiation alone did not induce p21 expression. There was no difference regarding p21 expression between wtp53 expressing cells with or without irradiation.

### DISCUSSION

In these experiments, we showed that wtp53 expression in SCC97 cells was associated with the restoration of p21 expression and a G1 arrest. However, when wtp53 was transferred immediately after irradiation, a prolonged G2 arrest was observed, with more than 80% of the cells arrested in G2, 48 h after treatment, followed by a massive apoptosis. This phenomenon was not related to the viral infection since neither G2/M arrest nor increased apoptosis were observed with the control null virus. In addition it was not due to γ-irradiation alone since the proportion of apoptotic cells following irradiation alone (about 30%) was much lower than when wtp53 was transferred after with irradiation (> 80%). Such a large increase of apoptotic cells after irradiation has also been observed in this histological type, using a protein kinase C inhibitor in combination with irradiation (Chmura et al, 1997). In addition, our results suggest that the timing of γ-irradiation before wtp53 gene transfer can be critical to obtain maximal effects in terms of apoptosis and cell killing. These observations could have important implications for future clinical protocols.

The wtp53-dependent G2 arrest observed in irradiated asynchronous cells was also observed in cells synchronized with mimosine, in the absence of irradiation. Indeed, when cells were released from the mimosine block, wtp53 induced a prolonged block in G2. This was also observed by Agarwal et al (1995) who showed in Li–Fraumeni syndrome fibroblasts expressing wtp53 under the control of the tetracycline promotor that expression of

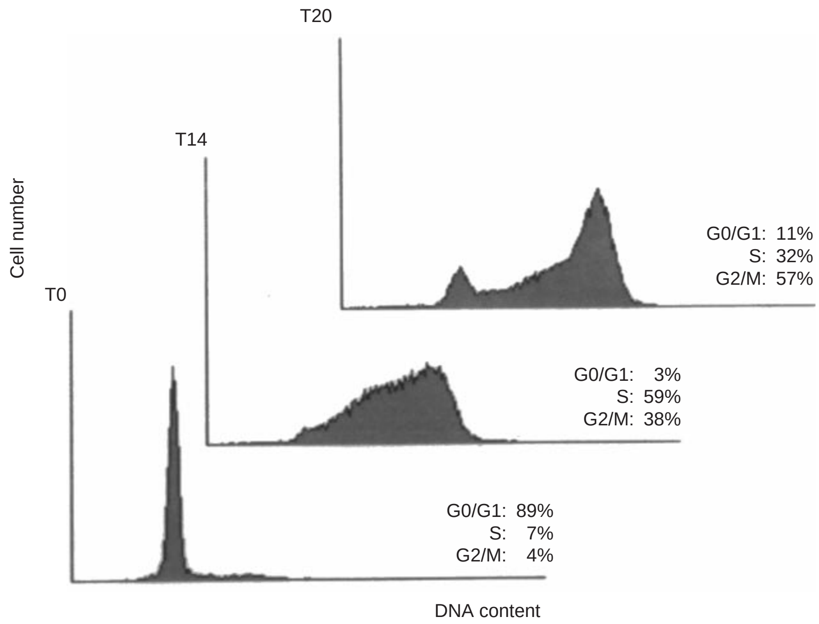

**Figure 3** Cell synchronicity: Cells were synchronized by mimosine for 24 h at 200 µM. Cells were released from the mimosine block at $t = 0$ and analysed by FACS at 0, 14 and 20 h by propidium iodine staining

*© 2000 Cancer Research Campaign*

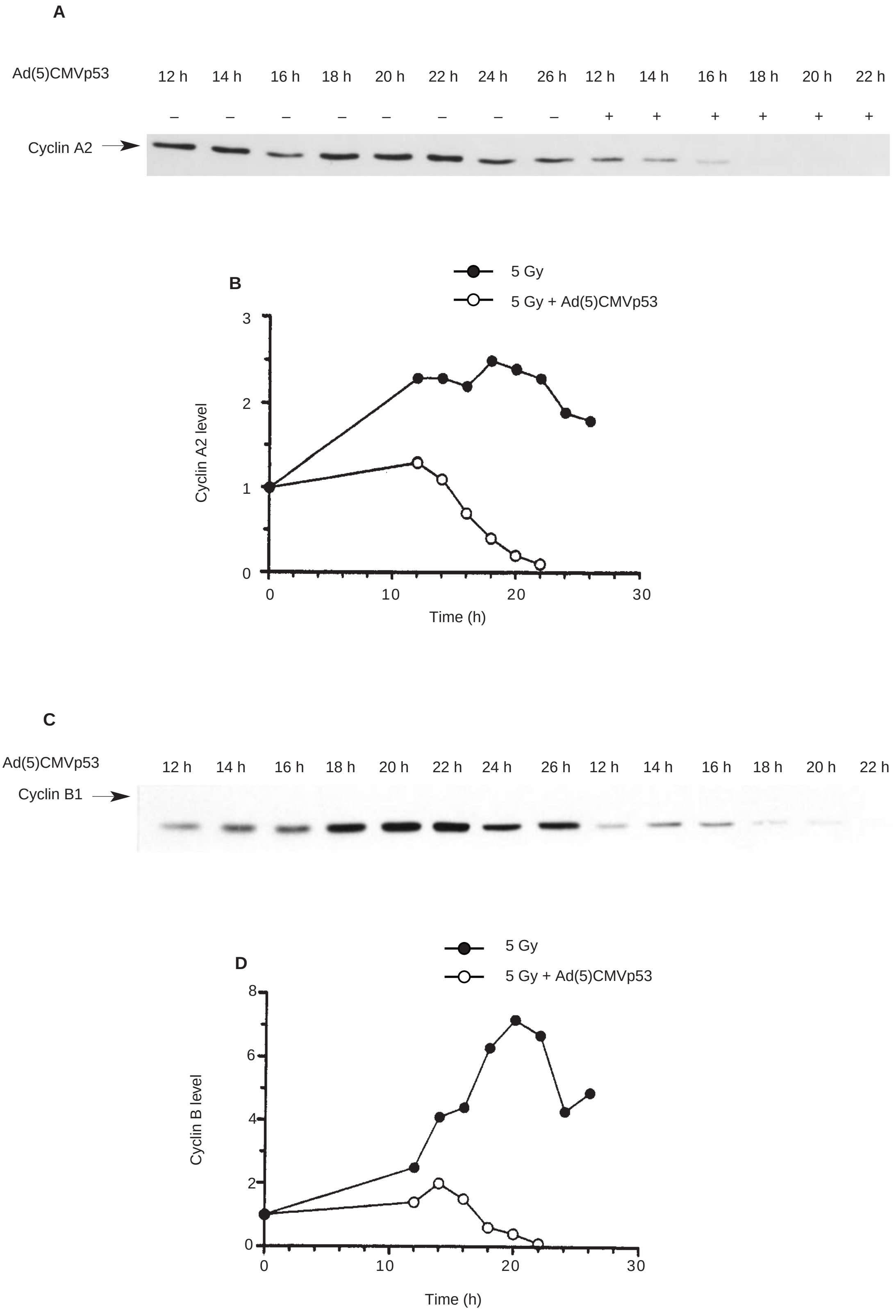

**Figure 4** Expression of cyclins A2 (**A**, upper part) and B1 (**B**, upper part) by Western blotting at different times after SCC97 cells were released from the mimosine block. Cells were irradiated with 5 Gy at *t* = 0. The hours represent the time after release from the mimosine block at *t* = 0. For each panel, the protein levels were determined by densitometric analysis of the Western blots. Results of densitometry scanning were presented as relative values (**A**, lower part and **B,** lower part)

*© 2000 Cancer Research Campaign*

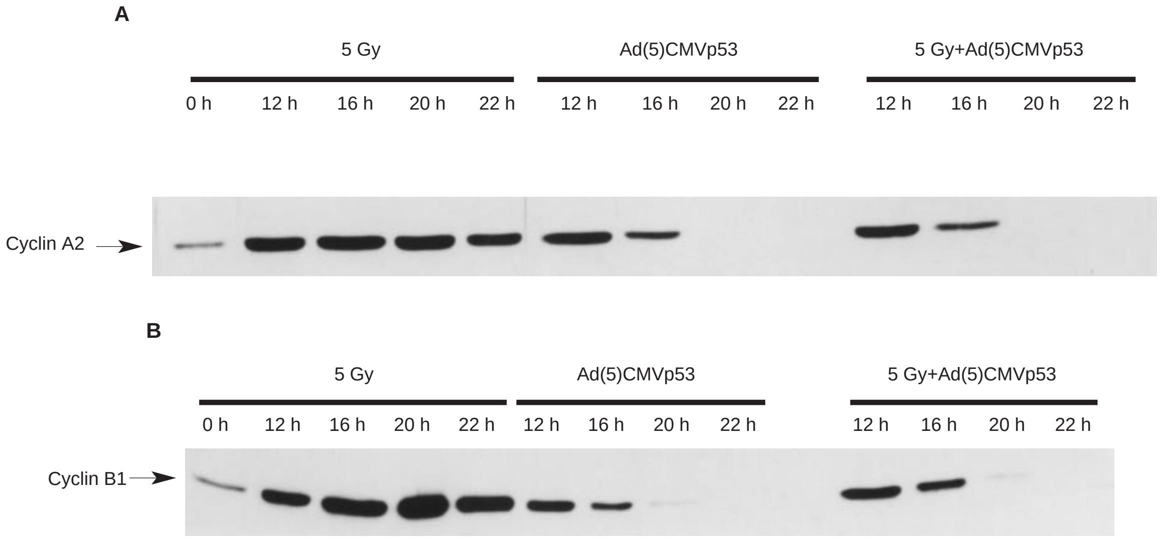

**Figure 5** Western blot analysis showing that the expression of wtp53 alone was sufficient to decrease the expression of both cyclins A2 and B1, without irradiation. Expression of cyclins A2 and B1 by Western blotting at different times after SCC97 cells were released from the mimosine block. The hours represent the time after release from the mimosine block at $t = 0$. Cells were irradiated with 5 Gy at $t = 0$

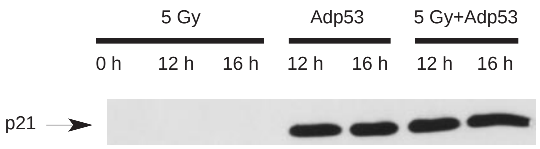

**Figure 6** p21 expression after irradiation alone and combined with Ad5CMV-*p53.* Cells were lysed and the proteins analysed by Western immunoblotting. Proteins, separated by SDS-PAGE, were transferred to a membrane and probed with antibodies recognizing p21 (Ab1)

wtp53 without any irradiation could mediate a predominant G2 arrest when cells were released from a mimosine block. In their case, wtp53 expression blocked the cells (for as long as 20 days) reversibly, in contrast with our results showing a G2 arrest followed by massive apoptosis. It should also be pointed out that the level of expression of the exogenous wtp53 following infection was probably much higher than the physiological one, since we used a strong CMV promotor. However, Agarwal et al (1995) showed that the G2/M arrest could be achieved in their model at physiological levels of p53. Our data suggest that the consequences of the p53-dependent G2 arrest can vary according to the model: massive apoptosis in our case, prolonged but reversible G2 arrest in Agarwal model and a third consequence has also been reported by Aloni-Grinstein et al (1995) who showed that expression of wtp53 was able to mediate differentiation during the G2/M growth arrest in a murine pre-B-cell line.

The influence of p53 on the G2 arrest has also been observed by Powell et al (1995) who described a differential sensitivity of p53(–) and p53(+) cells to caffeine induced radiosensitization. They showed that there was a complete override of G2/M arrest in p53(–) cells, but no impact in p53(+) cells also suggesting that wtp53 may help cells to overcome the G2 arrest. In addition, Winters et al (1998) reported a p53-dependent pathway which could operate after exposure of human cells to ionizing radiation to promote G2 arrest accompanied by nuclear translocation rather than inhibitory phosphorylation of CDC2. Our results are in agreement with these different studies. Nevertheless we observed that wtp53 blocked the cells in G2/M and induced massive apoptosis of these cells, which was in contrast with the studies of Skladanowski et al (1997) and Guillouf et al (1995) who showed that wtp53 was not able to block the cells in G2/M but could promote the exit from the G2/M arrest induced respectively by γ-irradiation and etoposide and then was followed by apoptosis. However, these results were obtained with a murine myeloid leukaemia cell line which could have a different and specific behaviour since Han et al (1995) described an apoptosis p53-independent mechanism at the G2 checkpoint in cells after X-irradiation in HL-60 cells (human promyelocytic leukaemia).

The molecular mechanisms involved in the p53-regulated G2/M arrest are under investigation. A recent study by Hermeking et al (1997) has found that a protein called 14-3-3 sigma was a p53-regulated inhibitor of G2/M progression in a human cancer cell line. In our study, we analysed the molecular mechanisms of this G2/M arrest by studying cyclins A2 and B1, which are known to be implicated in the G2/M transition. Cells which arrived in G2 expressed cyclins A2 and B1 (after irradiation alone) with a maximal level delayed compared to the control. This was in agreement with Muschel et al (1993) who have shown that the expression of cyclin B1 was delayed in expression after irradiation. However, in their study, cyclin A and cyclin B expression responded differently to radiation since cyclin A2 had a peak of expression at the same time as the control.

Various hypotheses can be generated to explain the complete disappearance of cyclins A2 and B1 in our study. A first hypothesis is that the 14-3-3 sigma protein may interfere with the expression of cyclins A2 and B1, since this protein has been recently described as a determinant of p53-regulated G2/M arrest in a human cancer cell line (Hermeking et al, 1997). A second hypothesis is that the cyclins could be degraded by caspases, as suggested by Stack and Newport (1997) who described degradation of A-type cyclins by the ICE-like caspases in *Xenopus* embryo

 *© 2000 Cancer Research Campaign*

apoptotic cells. Indeed, in our experiment, the cyclin disappearance and G2 arrest were coincident with a massive apoptosis, favouring this hypothesis. A third possible explanation is related to the cell-cycle-regulated ubiquitin-protein ligase complex, known as cyclosome or anaphase-promoting complex (APC/C). It initiates exit from mitosis by degrading mitotic cyclins. The human *fizzy* gene (p55cdc) is the homologue of a *Drosophila* gene necessary for cyclin A and cyclin B degradation (Townsley and Ruderman, 1998). Up to now, very little is known about the APC/C pathway, the role played by the Fizzy/cdc20p protein family and how the machinery is inactivated in response to DNA damage. However, we could hypothesize that p53 plays a role on Fizzy transcriptional activation to prevent cells to exit from mitosis into interphase of the next cell cycle. Finally, wtp53 may repress transcription of both cyclins A2 and B1. In support for this hypothesis, Yamamoto et al (1994) showed that wtp53 was able to inhibit transcription from the cyclin A2 promoter.

P53 may also interfere with E2F activated transcription of the cyclin A2/B1. Indeed, recent observations have clearly shown that the transcriptional factor E2F played a determinant role in cell-cycle regulation and was able to interact with both p53 and MDM2 (Muller, 1995). In addition, Dimri et al (1996) have demonstrated that p21 suppressed the activity of E2F-responsive promoters as cdk1 and that E2F was a critical target and ultimate effector of p21 action in a retinoblastoma protein (pRB)-independent pathway. Moreover, it has been recently shown that p21, acting as a p53-dependent cdk inhibitor played a role at the G2/M transition by association with cyclin A–cdk and cyclin B–cdk and promoted a transient arrest in late G2 that could contribute to late cell cycle checkpoint controls (Dulic et al, 1998). A role of p21 in G2/M transition has also been reported by Niculescu et al (1998). Altogether, these data indicate that the decrease in cyclins A2 and B1 in cells transduced by wtp53 may result from either transcriptional inhibition or protein degradation. Immunoprecipitation of cyclins A2 and B1 after pulse-chase labelling revealed an initial inhibition of cyclins A2 and B1 expression followed by increased degradation (our unpublished data). These preliminary results therefore suggest that both transcriptional repression and protein degradation could play a role in cyclins A2 and B1 disappearance during the p53-mediated G2 arrest.

In conclusion, a protracted G2 arrest was observed when exponentially growing SCC97 cells were infected by Ad5CMV-*p53* immediately after γ-irradiation. This prolonged arrest was followed by massive apoptotic cell death. The possibility to obtain massive apoptosis, when delivering wtp53 gene immediately after irradiation, suggest that this therapeutic sequence could be relevant for future clinical protocols combining radiotherapy and wtp53 gene transfer.

The G2 arrest was also observed in the absence of irradiation in synchronized cells expressing wtp53, with a dramatic decrease in cyclins A2 and B1.

This study is the first to show a p53-dependent down-regulation of cyclins A2 and B1, thus providing a molecular basis for the wtp53-dependent G2 arrest. However, the molecular mechanisms involved in p53-mediated down-regulation of these mitotic cyclins need further investigations and may also vary from one cellular model to another.

### ACKNOWLEDGEMENTS

We thank Yann Lécluse (URA 1156 CNRS, Gustave-Roussy Institute) for help in establishing the flow cytometry analysis. Valérie Chambon Velasco and David Deblé (Gustave-Roussy Institute) are gratefully acknowledged for technical assistance.

### REFERENCES

Allday MJ, Inman GJ, Crawford DH and Farrel PJ (1995) DNA damage in human B cells can induce apoptosis, proceeding from G1/S when p53 is transactivation competent and G2/M when it is transactivation defective. *EMBO J* **14:** 4994–5005

Aloni-Grinstein R, Schwartz D and Rotter V (1995) Accumulation of wild-type p53 protein upon gamma-irradiation induces a G2 arrest-dependent immunoglobulin kappa light chain gene expression. *EMBO J* **14**: 1392–1401

Agarwal ML, Agarwal A, Taylor WR and Stark GR (1995) P53 controls both the G2/M and G1 cell cycle checkpoint and mediates growth arrest in human fibroblasts. *Proc Natl Acad Sci USA* **92**: 8493–8497

Azzam EI, de Toledo SM, Pykett MJ, Nagasawa H and Little JB (1997) CDC2 is down-regulated by ionizing radiation in a p53-dependent manner. *Cell Growth Differ* **8**: 1161–1169

Chmura SJ, Mauceri HJ, Advani S, Haimann R, Beckett MA, Nodzenski E, Quintans J, Kufe D and Weichselbaum R (1997) Decreasing the apoptotic threshold of tumor cells through protein kinase C inhibition and sphingomyelinase activation increases tumor killing by ionizing radiation. *Cancer Res* **57**: 4340–4347

Clayman G, El Naggar A, Lippman S et al (1998) Adenovirus-mediated p53 gene transfer in patients with advanced recurrent head and neck squamous cell carcinoma. *J Clin Oncol* **16**: 2221–2232

Deng C, Zhang P, Harper JW, Elledge SJ and Leder P (1995) Mice lacking p21 CIP1/Waf1 undergo normal development but are defective in G1 checkpoint control. *Cell* **82**: 675–684

Dimri GP, Nakanishi M, Desprez PY, Smith JR and Campisi J (1996) Inhibition of E2F activity by the cyclin-dependent protein kinase inhibitor p21 in cells expressing or lacking a functional retinoblastoma protein. *Mol Cell Biol* **16**: 2987–2997

Dulic V, Stein GH, Farahi Far D and Reed SI (1998) Nuclear accumulation of p21Cip1 at the onset of mitosis: a role at the G2/M-phase transition. *Mol Cell Biol* **18**: 546–557

Flaman JM, Frebourg T, Moreau V, Charbonnier F, Martin C, Chappuis P, Sappino AP, Limacher JM, Bron L, Tada M, Van Meir EG, Estreucher A and Iggo RD (1995) A simple p53 functional assay for screening cell lines, blood and tumors. *Proc Natl Acad Sci USA* **92**: 3963–3967

Friend S (1994) p53: a glimpse at the puppet behind the shadow play. *Science* **265**: 334–335

Gallardo D, Drazan K and McBride W (1996) Adenovirus-based transfer of wild-type p53 gene increases ovarian tumour radiosensitivity. *Cancer Res* **56**: 4891–4893

Guillouf C, Rosselli F, Krishnaraju K, Moustacchi E, Hoffman B and Liebermann DA (1995) p53 involvement in the G2 exit of the cell cycle: role in DNA damage-induced apoptosis. *Oncogene* **10**: 2263–2270

Han Z, Chatterjee D, He DM, Early J, Pantazis P, Wycke JH and Hendrickson EA (1995) Evidence for a G2 checkpoint in p53-independent apoptosis induction by X-irradiation. *Mol Cell Biol* **15**: 5849–5857

Hermeking H, Lengauer C, Polyak K, He TC, Zhang L, Thiagalingam S, Kinzler KW and Vogelstein B (1997) 14-3-3 sigma is a p53-regulated inhibitor of G2/M progression. *Mol Cell* **1**: 3–11

Jin P, Gu Y and Morgan DO (1996) Role of inhibitory CDC2 phosphorylation in radiation-induced G2 arrest in human cells. *J Cell Biol* **34**: 963–970

Kao GD, McKenna WG, Maity A, Blank K and Muschel RJ (1997) Cyclin B1 availability is a rate-limiting component of the radiation-induced G2 delay in HeLa cells. *Cancer Res* **57**: 753–758

Kastan MB, Onyekwere O, Sidransky D, Vogelstein B and Craig RW (1991) Participation of p53 protein in the cellular response to DNA damage. *Cancer Res* **51**: 6304–6311

© 2000 Cancer Research Campaign

Maity A, McKenna WG and Muschel R (1995) Evidence for post-transcriptional regulation of cyclin B1 mRNA in the cell cycle and following irradiation in HeLa cells. *EMBO J* **14**: 603–609
Muller R (1995) Transcriptional regulation during the mammalian cell cycle. *Trends Genet* **11**: 173–178
Muschel RJ, Zhang HB and McKenna WG (1993) Differential effect of ionizing radiation on the expression of cyclin A and cyclin B in HeLa cells. *Cancer Res* **53**: 1128–1135
Niculescu AB III, Chen X, Smeets M, Hengst L, Prives C and Reed ST (1998) Effects of p21Cip1/Waf1 at both the G1/S and the G2/M cell cycle transitions: pRb is a critical determinant in blocking DNA replication and in preventing endoreduplication. *Mol Cell Biol* **18**: 629–643
Pellegata NS, Antoniono RJ, Redpath JL and Stanbridge EJ (1996) DNA damage and p53-mediated cell cycle arrest: a reevaluation. *Proc Natl Acad Sci USA* **93**: 15209–15214
Powell SN, De Frank JS, Connell P, Eogan M, Preffer F, Dombkowski D, Tang W and Friend S (1995) Differential sensitivity of p53(–) and p53(+) cells to caffeine-induced radiosensitization and override of G2 delay. *Cancer Res* **55**: 1643–1648
Sherr CJ (1996) Cancer cell cycles. *Science* **274**: 1672–1677
Skladanowski A and Larsen AK (1997) Expression of wild type p53 increases etoposide cytotoxicity in M1 myeloid leukemia cells by facilitated G2 to M transition: implication for gene therapy. *Cancer Res* **57**: 818–823
Stack JH and Newport JW (1997) Developmentally regulated activation of apoptosis in xenopus gastrulation results in cyclin A degradation during interphase of the cell cycle. *Development* **124**: 3185–3195
Stewart N, Hicks GG, Paraskevas F and Mowat M (1995) Evidence for a second cell cycle block at the G2/M by p53. *Oncogene* **10**: 109–115
Townsley FM and Ruderman JV (1998) Proteolytic ratchets that control progression through mitosis. *Trends Cell Biol* **8**: 238–244
Winters ZE, Ongkeko WM, Harris AL and Norbury CJ (1998) p53 regulates cdc2 independently of inhibitory phosphorylation to reinforce radiation-induced G2 arrest in human cells. *Oncogene* **17**: 673–684
Yamamoto M, Yoshida M, Ono K, Fujita T, Ohtani-Fujita N, Sakai T and Nikaido T (1994) Effect of tumour suppressors on cell cycle-regulatory genes: RB suppresses p34cdc2 expression and normal p53 suppresses cyclin A expression. *Exp Cell* **210**: 94–101
Zhang WW (1995) Safety evaluation of Ad5CMV-p53 in vitro and in vivo. *Hum Gene Ther* **6**: 155–164

*© 2000 Cancer Research Campaign*
